# Buffalo Medical Association

**Published:** 1880-03

**Authors:** 


### SOCIETY REPORTS.

---

### BUFFALO MEDICAL ASSOCIATION.

*Stated Meeting, February 2, 1880.*

THE PRESIDENT, DR. LUCIEN HOWE, IN THE CHAIR.

AFTER the transaction of a certain portion of the routine business, the essay for the evening was read by Dr. J. W. Keene, whose subject was "Cæsarean Section." The history of the operation was given in detail, and special attention called to the first authentic records concerning it, which dated from the latter part of the fifteenth century. Referring to the results as reported by different authors, he said :

"A summary of the various statistics at command gives a mortality of 52.8 per cent. This includes the results of both champions and opponents, and even the estimate of Boerhaave, that only one mother in 14 survives the operation, and also the statement of Velpeau, that in England, out of 15 or 20 cases not one at the time of his writing had been successful."

The Cæsarean Section was then compared with embryotomy and the question discussed as to which was preferable in cases when mother and child were both living. The words of Osborn, Maureceau, Baudelocque and Smellie, were quoted as favorable to the former operation, while Caseaux and others were opposed to it. As usual, the advocates of the middle course, seem to be the wiser; a class, like Schroeder, prefer the Cæsarean Section, especially "when a living child can not be delivered by the natural passages, without sacrifice of its life and the mother desires the performance of the operation." Its general advantages were then pointed out by the writer who said, "we have to deal with healthy tissues in a reasonably healthy patient, and we always know what we shall find."

The special indications for its performance were when the fœtus could not be delivered alive or dead per vias naturales; surety in all cases where the smallest pelvis diameter is less than 15 lines, after rupture of the uterus, with cancer of the cervix greatly advanced, or when a mother volunteers to sacrifice her own life for the chances of saving the child.

In the discussion which followed:

Dr. Hartwig regretted that the writer could not fortify his statements by personal experience. As for himself he had on one occasion been obliged to attempt the extraction of a child from the uterus of a woman suddenly deceased, this being required under such circumstances by the laws of Germany, where he then resided. At that time he was surprised by the abundance of the hemorrhage, and thought some procedure might be adopted to prevent the entrance of the blood into the abdominal cavity. It would seem advisable to him, under such circumstances, to unite the walls of the uterus to the edge of the incision in the linea alba, by means of a few elastic stitches, and after that to proceed to the removal of the child.

Dr. Mynter thought that the results thus far reported from the operation of gastro-elytrotomy were so encouraging as to make it in every way preferable to the Cæsarean section. A brief review was given of the manner of procedure adopted by Professors Skene and Thomas, from which it appeared that the external incision was similar to that made in ligating the iliac artery, that through this the vagina was opened and the child brought into the world over the brim of the pelvis.

Dr. Kilburn called attention to a tabulated list of cases prepared with great care by Dr. Harris, of Philadelphia, which showed a total mortality to the mothers of about 56 per cent.

Under the head of voluntary communications Dr. Mynter read the history of a case of intestinal obstruction, which he attempted to relieve by an operation, and accompanied the paper by the presentation of an interesting post-mortem preparation. (See Clinical Reports.)

The case was discussed at some length by Drs. Keene, Hartwig and others, after which the Association adjourned.

